# The Influence of Cerebrospinal Fluid Abnormalities and APOE 4 on PHF-Tau Protein: Evidence From Voxel Analysis and Graph Theory

**DOI:** 10.3389/fnagi.2019.00208

**Published:** 2019-08-08

**Authors:** Yuan Li, Zhijun Yao, Yue Yu, Yu Fu, Ying Zou, Bin Hu, for the Alzheimer’s Disease Neuroimaging Initiative, Michael W. Weiner

**Affiliations:** ^1^School of Information Science and Engineering, Shandong Normal University, Jinan, China; ^2^School of Information Science and Engineering, Lanzhou University, Lanzhou, China

**Keywords:** PHF-Tau, graph theory, network properties, APOE 4, CSF-Tau

## Abstract

Mild cognitive impairment (MCI) is a transitional state between the cognitive changes in normal aging and Alzheimer’s disease (AD), which induces abnormalities in specific brain regions. Previous studies showed that paired helical filaments Tau (PHF-Tau) protein is a potential pathogenic protein which may cause abnormal brain function and structure in MCI and AD patients. However, the understanding of the PHF-Tau protein network in MCI patients is limited. In this study, 225 subjects with PHF-Tau Positron Emission Tomography (PET) images were divided into four groups based on whether they carried Apolipoprotein E ε4 (APOE 4) or abnormal cerebrospinal fluid Total-Tau (CSF T-Tau). They are two important pathogenic factors that might cause cognitive function impairment. The four groups were: individuals harboring CSF T-Tau pathology but no APOE 4 (APOE 4−T+); APOE 4 carriers with normal CSF T-Tau (APOE 4+T−); APOE 4 carriers with abnormal CSF T-Tau (APOE 4+T+); and APOE 4 noncarriers with abnormal CSF T-Tau (APOE 4−T−). We explored the topological organization of PHF-Tau networks in these four groups and calculated five kinds of network properties: clustering coefficient, shortest path length, *Q* value of modularity, nodal centrality and degree. Our findings showed that compared with APOE 4−T− group, the other three groups showed different alterations in the clustering coefficient, shortest path length, *Q* value of modularity, nodal centrality and degree. Simultaneously, voxel-level analysis was conducted and the results showed that compared with APOE 4−T− group, the other three groups were found increased PHF-Tau distribution in some brain regions. For APOE 4+T+ group, positive correlation was found between the value of PHF-Tau distribution in altered regions and Functional Assessment Questionnaire (FAQ) score. Our results indicated that the effects of APOE 4 and abnormal CSF T-Tau may induce abnormalities of PHF-Tau protein and APOE 4 has a greater impact on PHF-Tau than abnormal CSF T-Tau. Our results may be particularly helpful in uncovering the pathophysiology underlying the cognitive dysfunction in MCI patients.

## Introduction

Alzheimer’s disease (AD) is generally considered as a cognitive dysfunction, neurodegenerative disease. The clinical symptoms of AD patients include aggression, confusion, language breakdown and the loss of cognitive functions (Waldemar et al., 2007). Mild cognitive impairment (MCI) is regarded as the transition stage from normal aging to AD. More and more studies are concentrated on MCI (Dyrba et al., 2018). A previous study discovered that the paired helical Tau (PHF-Tau) protein was a highly disease-related factor in the brain that may induce the development of MCI (Cho et al., 2016). PHF-Tau is an attractive target for both MCI diagnosis and treatment (Chien et al., 2014). A prior study revealed that accumulations of PHF-Tau neuro tangles in olfactory bulb and nerve were found in all cases of definite AD (Arnold et al., 2010). Meanwhile, Tau in cerebrospinal fluid (CSF) and Apolipoprotein E ε4 (APOE 4) are all associated with increased risk of progression from MCI to dementia (Arnold et al., 2010) and they are the robust predictors of AD (Blom et al., 2009). CSF-Tau contains phosphorylated Tau (P-Tau) and total Tau (T-Tau), which are two important biomarkers of CSF in MCI patients. However, Zhang et al. (2018) pointed out that the impact of CSF P-Tau is not obvious enough on MCI. Therefore, we only paid attention to how the CSF T-Tau and APOE 4 affected on PHF-Tau network. The organization of metabolic networks in APOE 4 carriers indicated a less optimal pattern and APOE 4 might be a risk factor for MCI (Yao et al., 2015). It was pointed out that APOE 4 may increase susceptibility to molecular pathology and regulate the anatomical pattern of neurodegeneration in AD (Lehmann et al., 2014). CSF T-Tau is a crucial biomarker for detecting dementia: when positive CSF T-Tau were defined as values above the cut-off (≥320 ng/L), approximately 90% of people were found to have dementia (Mattsson et al., 2009). Although PHF-Tau, APOE 4, and CSF T-Tau are all the important disease-related factors, joint studies on these three factors are limited.

Imaging techniques offer an invaluable tool for assessing brain pathology *in vivo*. Within past decades, Magnetic Resonance Imaging (MRI) and Diffusion Tensor Imaging (DTI), as the modern brain mapping techniques, were used in the early diagnosis of MCI and AD, which have been widely utilized in different studies (Teipel, 2010; Salvatore et al., 2016). Functional connectivity, as measured with functional magnetic resonance imaging (fMRI), was diminished in AD (Sala-Llonch et al., 2015). Positron Emission Tomography (PET) is an advanced clinical imaging technology in nuclear medicine. The general method of PET is to inject a substance, such as glucose, protein, nucleic acid and fatty acid, short-lived radionuclides labeled (e.g., 18F, 11C, etc.) into the human body to detect the metabolic activity, the accumulation of the protein of human beings and so on. In recent years, 18F-AV-1451 PET (Tau-PET), a novel PHF-Tau tracer (previously known as T807), is demonstrated to detect tangle pathology *in vivo* (Marquié et al., 2015). It provides a new method to measure Tau neuronal tangles in the brain by *in vivo* neuroimaging. Cho et al. (2016) pointed out that the tracer AV-1451 PET could be used to effectively measure the content of PHF-Tau in AD and MCI.

Graph theory is a powerful framework that represents the brain as a complex network. It could provide a mathematical and conceptual framework to construct a whole network for exploring the topological patterns of brain networks (Sporns et al., 2004). Neural networks could be called “small-world” networks with densely local connectivity between neighboring regions and sparsely long-range connectivity among distant regions. Vecchio et al. (2015) proposed that MCI could affect the alterations of physiological and structural synapses in specific brain networks (Vecchio et al., 2015). In addition, brain networks established by graph theory were also applied to the disease prediction. Using brain networks, recent studies made great contributions to the prediction of ASD and AD (Zhao et al., 2018; Zheng et al., 2019). In recent years, using graph theory in the diagnosis and treatment of AD/MCI is still a popular method (Yao et al., 2018). However, whether these topological abnormalities emerge in PHF-Tau networks remain largely unknown.

In this study, we hypothesized that the PHF-Tau protein network affected by APOE 4 and abnormal CSF T-Tau would exhibit abnormal topological properties. By establishing the PHF-Tau network for the four groups introduced above, we attempted to investigate the alterations in topological patterns. Moreover, we sought to determine whether APOE 4 and abnormal CSF T-Tau would affect the distributions of PHF-Tan protein in the whole brain. Finally, we studied the Pearson correlation between the different distribution value of PHF-Tau protein and Functional Assessment Questionnaire (FAQ) scores. In conclusion, these findings may provide critical insight into the brain pathophysiological mechanism.

## Methodology and Materials

All AV-1451 PET images, MRI and CSF values were downloaded from the website of the Alzheimer’s Disease Neuroimaging Initiative (ADNI)^1^. The major goal of ADNI is to track the progression of AD by using biomarkers to assess the brain’s structure and function over the course of various states. The ADNI’s data are gathered from 50 sites in the United States and Canada. The ADNI was launched in 2003 by the National Institute on Aging, the National Institute of Biomedical Imaging and Bioengineering, the Food and Drug Administration, private pharmaceutical companies, and nonprofit organizations as a 5-year public–private partnership. The lead author of this initiative is Michael W. Weiner, MD, University of California, San Francisco (email ADNI: adni@loni.usc.edu). The data collections were proofread by each participating site’s Institutional Review Board. Diagnostic criteria are stipulated at http://adni.loni.usc.edu/study-design/#background-container.

### Subjects

**Figure 1:**
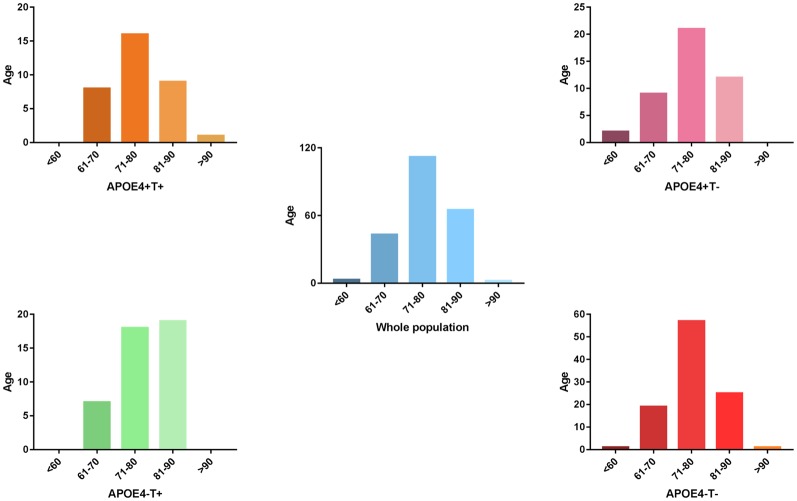
The distribution of whole population. We performed statistics according to the age distribution of the all subjects (age < 60; 61 < age < 70; 71 < age < 80; 81 < age < 90; age > 90). Blue histogram represents the age distribution of all subjects. Orange, purple, green, red histogram represent the age distribution of APOE 4+T+, APOE 4+T−, APOE 4−T+, APOE 4−T− group, respectively.

All the 225 subjects, including MCI and normal controls (NC; MCI:NC, 109:116), were divided into following four groups based on the value of T-Tau in CSF and APOE 4: (1) APOE 4 noncarriers and normal CSF T-Tau (APOE 4−T−; MCI:NC, 54:49); (2) APOE 4 carriers and abnormal CSF T-Tau (APOE 4+T+; MCI:NC, 16:18); (3) APOE 4 carriers and abnormal CSF T-Tau (APOE 4+T−; MCI:NC, 18:26); and (4) APOE 4 noncarriers and abnormal CSF T-Tau (APOE 4−T+; MCI:NC, 21:23). CSF T-Tau levels were considered abnormal if the CSF T-Tau were ≥320 ng/L, and the APOE information was obtained from the ADNI database. In ADNI database, MCI patients had reported a subjective memory concern either autonomously or through an informant or clinician. However, no significant level of impairment was present in other cognitive domains; essentially, their activities of daily living were preserved and no signs of dementia existed. The FAQ score is a bounded outcome with 0 indicating “no impairment” and 30 indicating “severe impairement.” The mini-mental state examination (MMSE) score can accurately reflect the mental state of the subjects. When the MMSE score is less than 27, it indicates that the subjects have MCI. The positive and negative status of Aβ and Tau protein are defined by Mattsson et al. (2009). Table 1 lists the demographic data of all subjects. The distribution of whole population could be found in Table 2 and Figure 1.

**Table 1:** Demographic data of all participants.

	APOE 4−T−	APOE 4+T+	APOE 4+T−	APOE 4−T+	APOE 4−T−vs.	APOE 4−T−vs.	APOE 4−T−vs.
	*N* = 103	*N* = 34	*N* = 44	*N* = 44	APOE 4+T+	APOE 4+T−	APOE 4−T+
Sex (male:female)	61:42	16:18	20:24	19:25	0.21^b^	0.12^b^	0.07^b^
Age	77.8 (6.5)	76.7 (7.1)	75.7 (7.1)	79.1 (7.0)	0.38^a^	0.09^a^	0.28^a^
FAQ	1.68 (4.24)	4.352 (6.75)	1.85 (5.01)	1.98 (5.55)	0.007^a^	0.85^a^	0.73^a^
MMSE	27.76 (1.87)	28.33 (1.55)	28.42 (1.41)	28.70 (1.35)	0.41^a^	0.54^a^	0.34^a^
CSF-Aβ (positive:negative)	90:13	30:4	37:7	38:6	0.92^b^	0.51^b^	0.75^b^
CSF-Tau (positive:negative)	9:94	31:3	8:36	37:7	<0.001^b^	0.12^b^	<0.001^b^
MCI:NC	54:49	16:18	18:26	21:23	0.58	0.45	0.60

*Data are represented as the mean (standard) deviations. ^a^P-value was obtained using the t-test. ^b^P-value was obtained using chi-square test*.

**Table 2:** The whole population distribution.

	All	APOE 4+T+	APOE 4+T−	APOE 4−T+	APOE 4−T−
<60	3	0	2	0	1
61–70	43	8	9	7	19
71–80	112	16	21	18	57
81–90	62	9	12	19	25
>90	2	1	0	0	1

Values of age and FAQ in Table 1 were reported as the mean (standard deviation) for each group. Group comparisons were performed using the chi-square or two-sample *t*-test (*P*-values).

### Data Acquisition and Preprocessing

MRI data were acquired on multiple 3 T MRI scanners using scanner-specific T1-weighted. The MRI data were preprocessed and further normalized to MNI standard space. PET scans required dynamic 30-min, six-frame (5-min each) acquisition starting 30 min after the injection of 18F-labeled AV-1451. The cross-sectional scan of MRI was chosen to be the closest in time to and acquired within 75 days on average of the Tau PET scan. AV1451-PET data were coregistered to the structural MRI data by using Statistical Parametric Mapping 12 (SPM12)^2^ running under MATLAB 2014a on the Centos 6.5 operating system. Then AV1451-PET data were corrected for partial volume effects. More details about the partial volume effects could be found in https://github.com/GGonEsc/petpve12.

CSF samples were obtained in the morning following an overnight fast from ADNI patients enrolled at 56 participating centers. A lumbar puncture was performed using a 20- or 24-gauge spinal needle. In brief, the CSF was collected into collection tubes provided at each site, transferred into polypropylene transfer tubes, frozen on dry ice within 1 h of collection, and shipped overnight to the ADNI Biomarker Core laboratory at the University of Pennsylvania Medical Center on dry ice. Aliquots (0.5 mL) were prepared from these samples after thawing (1 h) at room temperature and gentle mixing. The aliquots were stored in barcode-labeled polypropylene vials at 80°C. T-Tau and P-Tau levels were measured in each of the CSF baseline aliquots by using the multiplex xMAP Luminex platform (Luminex Corp, Austin, TX, USA) with Innogenetics (INNO-BIA AlzBio3; Ghent, Belgium; for research use-only reagents) immunoassay kit-based reagents (Shaw et al., 2009).

### Establishment of the Tau Network

In graph theory, a network is composed of nodes and edges that connect a vertices sequence. In our study, graph theory-based approaches were used to establish the PHF-Tau network of the four groups. A standardized automated anatomical labeling (AAL) template (90 brain regions in total, 45 in each cerebral hemisphere) was used to extract the brain regions. Subsequently, linear regression was performed to remove the effects of sex, age, and whole-brain PHF-Tau levels on patients’ measurements in each AAL brain region (Sanabria-Diaz et al., 2013). Three steps were adopted to estimate the correlation matrices. First, we used the linear regression model to remove the effects of age, gender and the average value of whole-brain PHF-Tau protein level for each subject. Second, a correlation matrix R with dimensions 90 × 90 was generated, where every individual entry *R*_ij_ was computed by the Pearson’s correlation coefficient between region i and j. Finally, the correlation matrices were obtained with diagonal elements equivalent to 1. The number of total probable correlations were 90× (90–1)/2 for each group. In the PHF-Tau networks, the nodes and edges corresponded to the AAL areas and the undirected connections of each pair in AAL areas, respectively. The topology of each group-network would differ significantly from each other under the thresholding of the same correlations value. In order to resolve this issue, sparsity (S) was used to threshold the correlations matrices of the networks into binarized matrices *P*, where an entry *P*_ij_ equals 1 if |*R*_ij_| exceeded sparsity and 0 otherwise. The binary matrices *P*_ij_ with N nodes and K edges were applied to simplify the PHF-Tau networks and reduce the computing scale for graph theory analysis. Sparsity was defined as the number of existing edges, K, divided by the maximum possible number of edges in a graph. There is no single optimal threshold selection method at present. We selected a sparsity value that ensures that all regions were included in the network while minimizing the number of false positive connections as usual. So that it can be used to threshold each group’s PHF-Tau network.

### Network Property Analysis

To explore the differences in PHF-Tau network properties between the APOE 4−T− group and the other three groups, we calculated the following indexes: network parameters of the clustering coefficient (Cp), characteristic path length (Lp), and *Q* value of modularity.

In graph theory, the average clustering coefficient of all nodes in the brain network measures the degree to which one node in a graph tends to cluster together. The characteristic path length is the average of the shortest paths of all pairs of nodes in the brain network, and it is a measure of the information efficiency or mass transport of a network (Stam et al., 2007). Notably, as the path length of the disconnected node is infinite, in this study, the harmonic mean path length (also called average inverse path length) was calculated as the characteristic path length.

We used the method of modularity for the PHF-Tau network as a parameter and the modularity separates into smaller communities, with dense links within itself and minor links between them (Newman and Girvan, 2004). The size of modularity for the PHF-Tau network as the aforementioned parameter is between the whole brain and a brain region, with close links within itself and subtle links between them (Newman, 2006). The quantity of modularity (Q) could be accurately obtained using the greedy agglomerative algorithm using the Brain Connectivity Toolbox (BCT; Rubinov and Sporns, 2010). The *Q* is a benchmark for quantifying community detection performance (Danon et al., 2005), and a high *Q* value indicates a robust division of the network.

### Nodal Properties

The “betweenness centrality” (BC) was defined as a local characteristic for exploring the outstanding nodes in the PHF-Tau networks. A node, which had a high betweenness might bridge different parts of the network (Rubinov and Sporns, 2010). BC is equal to the number of shortest paths from all vertices to all others that pass through that node. The hub nodes played a crucial role in facilitating information communication and processing among the human brain networks. BC is normally used to select candidate hubs in a network. The degree could express the number of network connections involving each node. The degree of all nodes is an important marker of brain network resilience.

All nodes properties were calculated using the BCT (Rubinov and Sporns, 2010). The properties value of each node was computed at a fixed sparsity 8% to ensure that the metabolic brain network was fully connected without fragmentation in each group.

### Statistical Analysis

A nonparametric permutation test was used to examine significant differences in PHF-Tau network properties, such as Cp, Lp, *Q* values of modularity, BC and degrees between the APOE 4−T− carriers and other three groups. In each case of fixed sparsity, the two groups’ properties were computed, pooled, and divided randomly into two randomized groups. In the test process, we maintained the number of sampled carriers as equal to the number in the original group. We computed differences in randomized groups, which was repeated 5,000 times. The same sparsity threshold was applied in each of the 5,000 cases, and we calculated the properties of each randomized group. Finally, the 5,000 recorded differences were sorted to determine the two groups’ differences in real PHF-Tau networks, which were included within 95% confidence interval (two-tailed) in the supposed between-group differences (Yao et al., 2010). In this permutation test, we selected the sparsity threshold values ranging from 8% to 30% for testing the significant between-group difference. This range of sparsity values could provide a fully connected undirected graph, which provided a reasonable estimation of the properties of networks (Stam et al., 2007). In the current situations, a fixed sparsity (*S* = 8%) could guarantee that all regions were included in the networks while minimizing the quantity of false-positive connections, thereby using this sparsity to threshold the PHF-Tau networks of each group. To explore the differences in PHF-Tau distribution among these four groups, the analysis of variance (ANOVA) and the *post hoc* test were used to determine differences between groups (APOE 4+T+, APOE 4+T−, APOE 4−T+ and APOE 4−T−).

First, ANOVA and *post hoc* test were performed in above four groups. Then, we get each significant different regions from ANOVA and *post hoc* test to form a mask for the three groups (APOE 4+T+, APOE 4+T− and APOE 4−T+). PHF-Tau values of corresponding brain regions were extracted using this mask for each subject in the above three groups. Finally, Pearson correlation analysis was conducted using extracted PHF-Tau values and FAQ scores in each group. Our results showed that only APOE 4+T+ group have positive correlation with FAQ scores. We also explored the regional and global correlations between CSF Tau and Tau-PET.

## Results

No significant differences were found in age or sex among the four groups in demographic characteristics. However, FAQ differed significantly between the APOE 4+T+ and APOE 4−T− (*P* < 0.05). The Aβ-positive and Aβ-negative were controlled. Since the subjects grouping were based on the content of CSF T-Tau, there was significant difference between the Tau-positive and Tau-negative (Table 1). We performed statistics according to the age distribution of the all subjects (age < 60; 61 < age < 70; 71 < age < 80; 81 < age < 90; age > 90; Figure 1 and Table 2).

### Comparison of PHF-Tau in the Brain

Compared with the APOE 4−T− group, the APOE 4+T+ group showed increased distribution of PHF-Tau protein in some regions, and details were shown in Table 3 (*P* < 0.05; FDR-corrected).

**Table 3:** Differences in regions in terms of Tau distribution in the APOE 4+T+ group.

AAL brain region	Side	MNI coordinate			*F*-value
		*X*	*Y*	*Z*	
ParaHippocampal	Left	−25	−20	−10	7.84
ParaHippocampal	Right	29	19	10	6.37
Amygdala	Left	−23	−0.6	−17	8.89
Fusiform	Left	−31	−40	−20	11.23
Fusiform	Right	32	−39	−20	7.44
Temporal_Sup	Right	58	−21	7	14.48
Temporal_Mid	Left	−55	−33	−2	9.55
Temporal_Mid	Right	57	−37	−1	6.74
Temporal_Inf	Left	−50	−28	−23	7.29
Temporal_Inf	Right	52	−31	−22	6.86

Compared with the APOE 4−T− group, the APOE 4+T− group, the increased PHF-Tau regions are shown in Table 4 (*P* < 0.05; FDR-corrected).

**Table 4:** Differences in regions in terms of Tau distribution in the APOE 4+T− group.

AAL brain region	Side	MNI coordinate			*T*-value
		*X*	*Y*	*Z*	
Amygdala	Left	−18	−4	−13	3.36
ParaHippocampal	Right	26	20	13	3.39
Occipital_Inf	Left	−30	−87	−7	3.43
Temporal_Sup	Right	58	−17	6	3.29
Temporal_Mid	Left	−52	−36	−3	3.32

Finally, a comparison of the APOE 4−T+ group with the APOE 4−T− group showed that the left middle frontal gyrus was the only increased region (*P* < 0.05; FDR-corrected).

### Correlation Analysis

**Figure 2:**
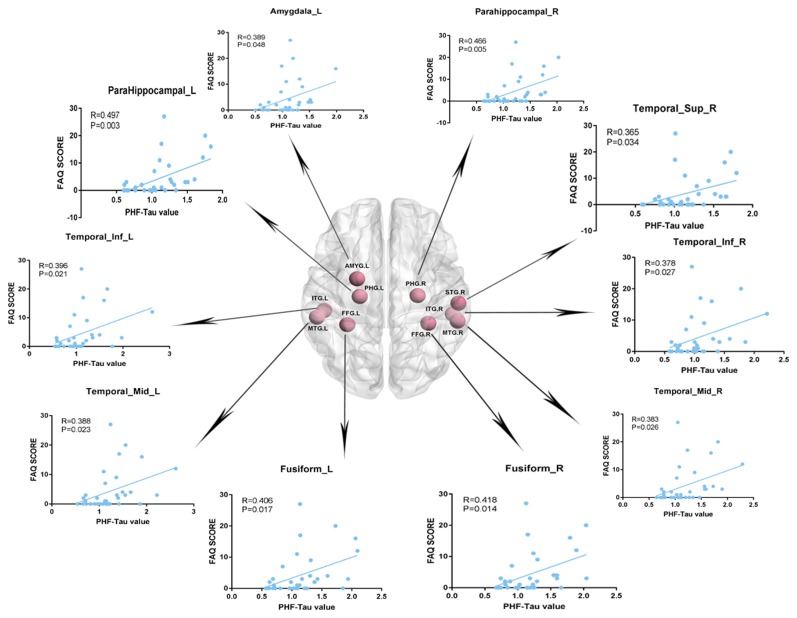
Pearson correlation analysis between Functional Assessment Questionnaire (FAQ) scores and increased regions of paired helical filaments-Tau (PHF-Tau) value comparing the APOE 4+T+ group with the APOE 4−T− group (*P* < 0.05, FDR). Pink nodes represented the different significant region, and the Pearson correlation analysis results of the corresponding regions were marked by arrows.

We analyzed the Pearson correlation between FAQ score and value of PHF-Tau distribution in altered regions in the three groups. A positive correlation was found between all the regions with significant differences and FAQ score in APOE 4+T+ group, while there was no significant correlation in the other two groups. Specific correlation results were shown in Figure 2 and Table 5.

**Table 5:** Pearson correlation analysis (FDR, *P* < 0.05).

Brain region	FAQ *P*-value	FAQ *R*-value
ParaHippocampal_L	0.003	0.497
ParaHippocampal_R	0.005	0.466
Amygdala_L	0.048	0.389
Fusiform_L	0.017	0.406
Fusiform_R	0.014	0.418
Temporal_Sup_R	0.034	0.365
Temporal_Mid_L	0.023	0.388
Temporal_Mid_R	0.026	0.383
Temporal_Inf_L	0.021	0.396
Temporal_Inf_R	0.027	0.378

We also calculated the Pearson correlation between CSF Tau and PET. Results showed the significant correlations between PHF-Tau value of left olfactory bulb and CSF T-Tau, PHF-Tau value of right parahippocampal gyrus and CSF T-Tau in the APOE 4+T+ group. The detailed results were showed in the Supplementary Materials.

### Network Properties

**Figure 3:**
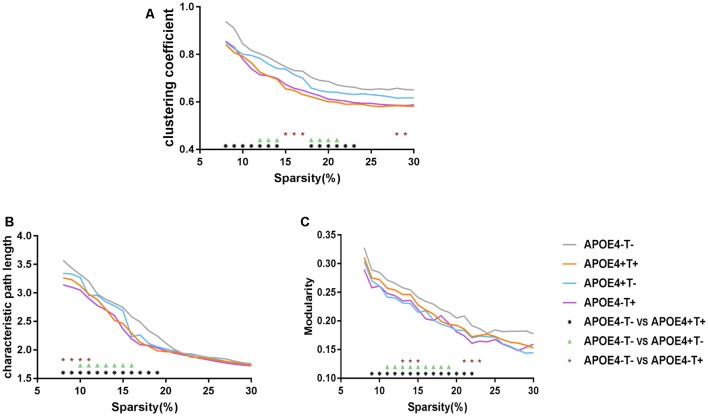
Network properties. Mean values for the clustering coefficient **(A)**, characteristic path length **(B)** and modularity **(C)** for APOE 4−T− (gray), APOE 4+T+ (orange), APOE 4+T− (blue), APOE 4−T+ (purple) at different network densities. Black asterisk indicates a statistically significant difference between APOE 4−T− and APOE 4+T+. Green triangle indicates a statistically significant difference between APOE 4−T− and APOE 4+T−. Brown star indicates a statistically significant difference between APOE 4−T− and APOE 4−T+.

Compared with APOE 4−T−, the other three groups showed significant differences in the three network properties: Cp, Lp, and Q. The APOE 4+T+ group showed lower Cp (sparsity ranges: 8%–14% and 18%–23%; Figure 3A), Lp (sparsity = 8%–19%; Figure 3B), and *Q* value (sparsity = 9%–22%; Figure 3C). In the APOE 4+T− group, the significant descreased difference was observed in Cp (sparsity = 12%–14% and 18%–21%; Figure 3A), Lp (sparsity = 10%–16%; Figure 3B) and *Q* (sparsity 11%–19%; Figure 3C). And APOE 4−T+ group showed significant decreased difference in Cp (sparsity = 15%–17% and 29%–30%; Figure 3A), Lp (8%–11%; Figure 3B), and *Q* value (sparsity = 13%–15% and 21%–23%; Figure 3C).

### Nodal Properties

**Figure 4:**
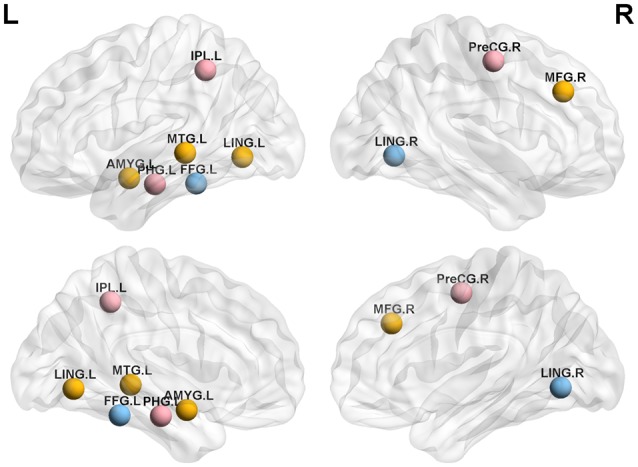
Nodal centrality of brain network. Results showed significantly different regions in the nodal centrality between APOE 4−T− and the following groups: APOE 4+T+ (orange nodes), APOE 4+T− (pink nodes), APOE 4−T+ (blue nodes), after 5,000 permutation testing (*p* < 0.05, FDR-corrected). Abbreviation: IPL.L, left inferior parietal, but supramarginal and angula gyri; PHG.L, left parahippocampal gyrus; LING.L, left lingual gyrus; MTG.L, left middle temporal gyrus; AMYG.L, left amygdala; FFG.L, left fusiform gyrus; PreCG.R, right precentral gyrus; MFG.R, right Middle frontal gyrus; LING.R, right lingual gyrus.

We analyzed the nodal centrality and the degree of each node. Nodal centrality showed significant differences in some regions for the APOE 4+T+ group, compared with the APOE 4−T− group (Figure 4 and Table 6). The increased regions were observed in the right middle frontal gyrus, left lingual gyrus, whereas decreased regions were observed in the left middle temporal gyrus, left amygdala. Compared with the APOE 4−T− group, the APOE 4+T− group showed increased regions which were in the left inferior parietal (but supramarginal and angula gyri), right precentral gyrus whereas the decreased region was in left parahippocampal gyrus (Figure 4 and Table 6). The results also showed significant nodal centrality differences for the APOE 4−T+ group compared with the APOE 4−T− group (Figure 4, Table 6). The increased regions were located in the left fusiform gyrus and right lingual gyrus.

**Table 6:** Significant differences in the hub regions (*P* < 0.05; FDR-corrected).

Region	APOE 4−T−	APOE 4+T+	*P*-value
Frontal_Mid_R	0.1	3.77	*P* < 0.001
Temporal_Mid_L	5.27	2.15	*P* < 0.001
Amygdala_L	3.33	1.01	*P* < 0.001
Lingual_L	0.21	4.38	*P* = 0.003
Parahippocampal_L	5.39	1.24	*P* < 0.001
Parietal_Inf_L	0.51	4.36	*P* = 0.01
Precentral_R	0.48	2.84	*P* < 0.001
Fusiform_L	0.03	3.55	*P* < 0.001
Lingual_R	0.27	3.82	*P* < 0.001

**Figure 5:**
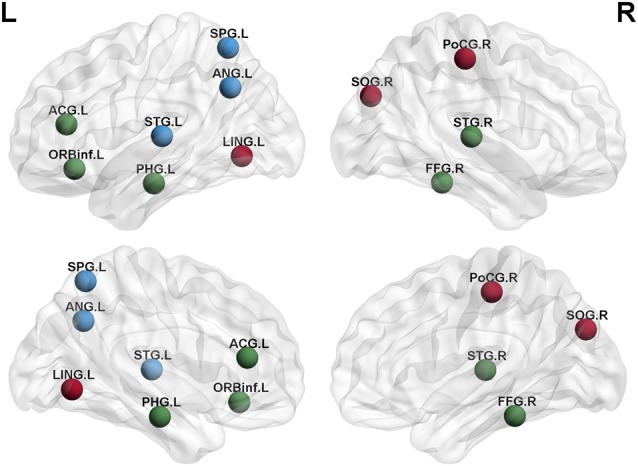
Nodal degree of brain network. Results showed significantly different regions in the degree between APOE 4−T− and the following groups: APOE 4+T+ (green nodes), APOE 4+T− (brown nodes), APOE 4−T+ (blue nodes), after 5,000 permutation testing (*p* < 0.05, FDR-corrected). Abbreviation: SPG.L, left superior parietal gyrus; ANG.L, left angular gyrus; STG.L, left superior temporal gyrus; ACG.L, left anterior cingulate and paracingulate gyri; ORBinf.L, left inferior frontal gyrus (orbital part); PHG.L, left parahippocampal gyrus; LING.L, left lingual gyrus; SOG.R, right superior occipital gyrus; PoCG.R, right postcentral gyrus; STG.R, right superior temporal gyrus; FFG.R, right fusiform gyrus.

Degree analysis showed significant differences in some regions for the APOE 4+T+ group, compared with the APOE 4−T− group (Figure 5 and Table 7). The increased regions were observed in the right superior temporal gyrus, and left inferior frontal gyrus (orbital part), whereas decreased regions were observed in the left anterior cingulate and paracingulate gyri, right fusiform gyrus, and left parahippocampal gyrus. Compared with the APOE 4−T− group, the APOE 4+T− group showed increased regions which were in the right superior occipital gyrus, left lingual gyrus whereas the decreased region was in the right postcentral gyrus (Figure 5 and Table 7). The results also showed significant degree differences for the APOE 4−T+ group compared with the APOE 4−T− group (Figure 5, Table 7). The decreased regions were located in the left angular gyrus, left superior parietal gyrus and left superiortemporal gyrus.

**Table 7:** Significant differences in the degrees (*P* < 0.05; FDR-corrected).

Region	APOE 4−T−	APOE 4+T+	*P*-value
Temporal_Sup_R	0.60	1.17	*P* < 0.001
Frontal_Inf_Orb_L	−0.20	1.28	*P* = 0.004
Cingulum_Ant_L	−0.61	−2.05	*P* = 0.009
Fusiform_R	0.95	0.23	*P* = 0.005
Parahippocampal_L	1.73	0.25	*P* < 0.001
Occipital_Sup_R	0.88	1.73	*P* = 0.01
Lingual_L	0.91	2.77	*P* = 0.009
Postcentral_R	3.55	0.48	*P* < 0.001
Angular_L	1.02	0.36	*P* < 0.001
Parietal_Sup_L	1.73	0.19	*P* = 0.03
Temporal_Sup_L	2.55	1.34	*P* < 0.001

## Discussion

In this study, we examined the topological patterns of the PHF-Tau network using the AV-1451-PET data. To the best of our knowledge, this is the first research to explore the PHF-Tau protein network affected by abnormal CSF T-Tau and APOE 4. The main findings of our study could be summarized as: (1) Voxel level analysis suggested that compared with the APOE 4−T− group, the other three groups (APOE 4+T+, APOE 4+T− and APOE 4−T+) showed increased distribution of PHF-Tau protein in several regions; (2) In APOE 4+T+ group, the positive correlation between value of PHF-Tau distribution in altered regions and FAQ score was obtained; (3) APOE 4 and abnormal CSF T-Tau have affected on the topological structure of PHF-Tau protein network, and the impact of APOE 4 is more obvious; and (4) Compared with APOE 4−T− group, the other three groups showed significantly different regions in nodal properties.

### Comparison in Whole Brain PHF-Tau and Correlation Analysis

Changes in the distribution of PFH-Tau were found between APOE 4−T− and other three groups. Results showed that the distribution of PHF-Tau protein was significantly increased in the bilateral temporal lobe, left amygdala, bilateral fusiform, and bilateral parahippocampal gyrus in the APOE 4+T+ group and the increased distribution of PHF-Tau protein was distributed in the left amygdala, right parahippocampal gyrus, bilateral temporal lobe and left occipital lobe in APOE 4+T− group, while the significant increased distribution of PHF-Tau was only found in the left middle frontal gyrus for APOE 4−T+ group. Meanwhile, the results of regional correlations between CSF T-Tau and PET revealed the significant correlations in the right parahippocampal gyrus and left olfactory bulb in APOE 4+T+ group. A previous study found an increased neurofibrillary tangle number in the fusiform cortex was influenced by APOE 4 (Beffert and Poirier, 2010) and the abnormal CSF Tau could affect the cortical thickness of the fusiform (Fortea et al., 2014). Owing to fusiform cortex was related to the visual function of the brain, MCI patients with abnormal fusiform cortex might have visual recognition deficits. A previous study showed temporal lobe which was closely related to sensory function had an increased Tau content (Schöll et al., 2017). Additionally, the left temporal cortex was considered to be a hub for semantic processing for object names (Tsapkini et al., 2011). APOE 4 carriers were consistent with abnormalities on neuroimaging in AD patients, and severe atrophy of the medial temporal lobe was reported in patients with MCI and AD (Filippini et al., 2009). Furthermore, CSF-Tau was correlated with impairment of glucose metabolism in temporal lobes, posterior cingulate, and the superior parietal gyrus and precentral gyrus (Haense et al., 2008). Observed by Rubinov and Sporns (2010), a significant negative correlation between the gray matter density of the parahippocampus and CSF-Tau levels in MCI patients. In pre-symptomatic Alzheimer patients, the lack of connectivity in the default mode network (DMN) primarily in the parahippocampal gyrus among APOE 4 carriers was found (Goryawala et al., 2015). APOE 4 might interfere with synaptogenesis during memory processing, so the hippocampal and amygdala structures of different APOE genotypes in AD patients were damaged to varying degrees (Lehtovirta et al., 1995). Moreover, the increased distribution of PFH-Tau in occipital lobe was only in APOE 4+T− group and the previous study proved that the presence of APOE 4 allele might affect the activity of choline acetyltransferase in the brain of AD, including hippocampus, temporal and occipital cortex (Allen et al., 1997). Besides, Liu et al. (2013) suggested occipital dementia had its own neuropsychological phenotype which may be a type of AD. A prior study found the middle frontal gyrus is associated with executive ability (Unterrainer and Owen, 2006). Decreased white matter volume and abnormal signal in the frontal lobe in AD and MCI patients may lead to cognitive abnormalities in the brain (Leritz et al., 2014). The olfactory bulb is an essential brain region that manages the human olfactory system, which is destroyed in the process of dementia (van der Berg et al., 2007). A previous study suggested that olfactory dysfunction occurs early in many neurodegenerative diseases, particularly of Parkinson’s disease, MCI and AD (Attems et al., 2014). We could speculate that the abnormality of PHF-Tau might lead to the decline in execution control ability.

We assessed the Pearson correlation between the value of PHF-Tau distribution in altered regions and FAQ score in APOE 4+T+ group. The results showed that in the APOE 4+T+ group, all regions with increased PHF-Tau protein distribution were positively correlated with FAQ scores. This suggested that the participants with high FAQ score might be due to the increased distribution of PHF-Tau protein, resulting in clinical symptoms such as the poor mental status and more severe dementia. In congruence with previous studies, our findings provided additional evidence that increased distribution of PHF-Tau protein in individuals was related to severe cognitive impairment (Weaver et al., 2000). The Pearson correlation between FAQ score and APOE 4+T+ group indicated that APOE 4 and CSF T-Tau were both possible factors for dementia.

### Network Properties Analysis of the PHF-Tau Network

Regarding global network topology, we found widespread changes in integration measures in APOE 4+T+, APOE 4+T−, and APOE 4−T+ individuals. These changes were in the clustering coefficient, path length, and *Q* value of modularity. The clustering coefficient was based on correlations between adjacent brain regions, which reflected the degree of node aggregation (Seo et al., 2013). Because the ability of Tau plaque transmission was significantly stronger in the three abnormal groups, Tau plaques could easily spread to remote brain regions. As a result, this phenomenon was reflected at the network level. The three groups exhibited a lower clustering coefficient and a weaker local specialization which suggested that Tau plaques aggregated together and transported to different brain regions easily (Duan et al., 2017). Path length measures the ability of information transmission across brain regions. A low path length indicated a shorter information transmission path between two nodes (Sporns and Zwi, 2004). In patients, Tau plaques could spread to other remote brain regions. As a result, although two regions were far away from each other, their correlation might be strong. At the network level, a strong correlation means that connection exists between the regions. Therefore, shorter paths could be achieved from one node to another remote node, eventually causing abnormal groups of subjects to exhibit a lower path length. This suggested that shorter Tau transmission paths could be observed between two brain regions in the three abnormal groups. Relevant studies combining graph theory with DTI (Lo et al., 2010), structural MRI (Pereira et al., 2016), functional MRI (Zhao et al., 2012), and electroencephalogram data (Stam et al., 2007) have found similar changes in patients with AD. Meanwhile, the alterations of *Q* value in modularity were found in the present study. The graph measure reflects the ability of the network to process information within clusters of regions or modules (Haense et al., 2008). Modularity assesses the extent to which a network can be divided into smaller communities of regions or modules that potentially share a specific function (Newman and Girvan, 2004). Modularity is a parameter to illustrate how to divide brain networks into smaller communities, with maximal links within itself and minimal links among the communities (Cope et al., 2018; Pereira et al., 2018). The *Q* value of modularity quantitatively measures the quality of each node assigned to the communities (Newman and Girvan, 2004). Additionally, previous studies have found that the clustering coefficient was an indicator of modularity, which showed differences between AD patients and normal people (Cope et al., 2018). The abnormalities of APOE 4 and CSF T-Tau would induce the change of PHF-Tau distribution, thus affecting the state of modularity. Changes found in this study may indicate that the distribution of Tau protein is more extensive, making local connections closer.

### Abnormal Changes in Nodal Properties

Figures 4, 5 showed that the significant abnormal nodal properties were mainly distributed in frontal lobe, occipital lobe (lingual gyrus), left parietal lobe, right precentral gyrus, bilateral fusiform gyrus, bilateral temporal lobe, left amygdala, left parahippocampal gyrus, left anterior cingulate gyrus, right postcentral gyrus and left angular gyrus. Since the frontal lobe has been interpreted as being closely related to memory and semantic processing in the brain, the changed nodal properties in the frontal lobe may cause memory impairment (Gabrieli et al., 1996). Fennema-Notestine et al. (2011) suggested that the APOE 4 allele might affect cortical thickness in frontal regions. Increased Tau protein levels in occipital had a significant pathological correlation with MCI and AD and were also strongly correlated with the Braak stage (Schwarz et al., 2016). APOE 4 carriers showed significantly decreased functional connectivity in brain regions, which involved in learning and memory functions, including frontal lobe and basal ganglia regions (Li et al., 2014). A prior study reported the parietal area of APOE 4 carriers showed significant abnormal volume changes compared with non-carriers (Honea et al., 2009). APOE 4 carriers showed increased interregional correlation coefficients in precentral gyrus (Goryawala et al., 2015). Cai et al. (2015) demonstrated that MCI patients had widespread alterations in fusiform connectivity using task fMRI. As for FDG-PET metabolism, some studies showed that metabolism decreased, especially in the posterior parietal lobe of AD-sensitive region, and worsened with the increase of the number of APOE 4 alleles (Lehmann et al., 2014). Metabolic dysfunction was most common in the temporal and parietal lobes associated areas, and metabolic decline in the medial parietal lobes helped to distinguish from AD patients in control participants more accurately (Villain et al., 2010). Several studies reported that MCI patients presented with episodic memory impairment, which led to global cognitive impairment with the disease progresses. Neuroimaging showed medial temporal lobe atrophy in the early stage of the disease, while generalized temporal lobe and whole-brain atrophy were the characteristics of late AD (Fox et al., 1996). The amygdala is involved in emotional memory, attention, perception and so on (Phelps, 2006). Many studies found significant atrophy of the amygdala in MCI/AD patients (Poulin et al., 2011). APOE 4 allele is thought to cause structural abnormalities in the amygdala of MCI patients (Lehtovirta et al., 1996). A previous pathological aging study showed that parahippocampal volume as a biomarker had a better discriminatory effect than the volume of the hippocampus in differentiating MCI group from healthy control group (de Toledo-Morrell et al., 2010). The APOE 4 carriers exhibit increased connectivity and functional-activation changes in the cingulate cortex (Chen et al., 2007). Atrophy of the anterior cingulate cortex showed the greatest correlation with verbal episodic memory deficit in MCI patients (Leube et al., 2008). A prior study reported that the abnormal gray matter volume was in angular gurus of the MCI group (Gispert et al., 2016). Haense et al. (2008) found the lower MMSE score was significantly correlated with the decrease of metabolism in the postcentral gyrus. Aforementioned changes might explain the effects of APOE 4 and CSF-Tau on MCI and even AD at a deeper level. Through the above analysis, we found that APOE 4 and CSF T-Tau affected regions might not only be related to MCI, but also an important factor in transforming MCI into AD. These results showed APOE 4 has a greater impact on PHF-Tau than abnormal CSF T-Tau.

## Conclusion

In this study, we combined the voxel level analysis and graph theory approach to investigate the PHF-Tau protein distribution and the alterations of PHF-Tau protein networks topology organization affected by APOE 4 and abnormal CSF T-Tau proteins. The results suggested that compared with APOE 4−T− group, the other three groups exhibited alterations in PHF-Tau protein distribution and losses of network properties. Additionally, we found that APOE 4 may be a more significant factor for PHF-Tau protein relative to CSF T-Tau in the brain. Finally, the simultaneous effects of APOE-4 and abnormal CSF T-Tau may aggravate the decline of FAQ score. Findings in this study might elucidate the pathological mechanism of MCI and AD for further perspective studies.

## Limitation

The present study had two limitations. First, some of the moderating variables, such as average education years and intelligence quotient, were not collected in this analysis. Second, the study lacked participants with AD, which made the analysis incompletely. Lastly, it should be noted that the AV-1451 combination mainly recognizes the aggregated Tau in the tangles and does not measure the oligomeric Tau protein.

## Data Availability

Publicly available datasets were analyzed in this study. This data can be found here: http://adni.loni.usc.edu.

## Ethics Statement

The study procedures were approved by the institutional review boards of all participating centres (https://adni.loni.usc.edu/wp-content/uploads/how_to_apply/ADNI_Acknowledgement_List.pdf), and written informed consent was obtained from all participants or their authorised representatives. The investigators within the ADNI contributed to the design and implementation of the ADNI and/or provided data but did not participate in analysis or writing of this report.

## Author Contributions

YL, BH and ZY conceived and designed the experiments. YL, BH, ZY, YY, YF and YZ analyzed the data. YL, BH, ZY, YF and YZ contributed reagents, materials and analysis tools. YL, BH, ZY and YZ wrote the article.

## Conflict of Interest Statement

The authors declare that the research was conducted in the absence of any commercial or financial relationships that could be construed as a potential conflict of interest.

## Footnotes

^1^http://adni.loni.usc.edu/

^2^https://www.fil.ion.ucl.ac.uk/spm/software/spm12/

## References

[B1] Allen S. J. MacGowan S. H. Tyler S. Wilcock G. K. Robertson A. G. S. Holden P. H.. (1997). Reduced cholinergic function in normal and Alzheimer’s disease brain is associated with apolipoprotein E4 genotype. Neurosci. Lett. 239, 33–36. 10.1016/s0304-3940(97)00872-0 9547165

[B2] Arnold S. E. Lee E. B. Moberg P. J. Stutzbach L. Kazi H. Han L.-Y.. (2010). Olfactory epithelium amyloid-β and paired helical filament-tau pathology in Alzheimer disease. Ann. Neurol. 67, 462–469. 10.1002/ana.21910 20437581 PMC2864948

[B3] Attems J. Walker L. Jellinger K. A. (2014). Olfactory bulb involvement in neurodegenerative diseases. Acta Neuropathol. 127, 459–475. 10.1007/s00401-014-1261-7 24554308

[B4] Beffert U. Poirier J. (2010). Apolipoprotein E, plaques, tangles and cholinergic dysfunction in Alzheimer’s disease. Ann. N Y Acad. Sci. 777, 166–174. 10.1111/j.1749-6632.1996.tb34415.x 8624080

[B5] Blom E. S. Giedraitis V. Zetterberg H. Fukumoto H. Blennow K. Hyman B. T.. (2009). Rapid progression from mild cognitive impairment to Alzheimer’s disease in subjects with elevated levels of tau in cerebrospinal fluid and the *APOE* ε4/ε4 genotype. Dement. Geriatr. Cogn. Disord. 27, 458–464. 10.1159/000216841 19420940 PMC7077080

[B6] Cai S. Chong T. Zhang Y. Li J. von Deneen K. M. Ren J.. (2015). Altered functional connectivity of fusiform gyrus in subjects with amnestic mild cognitive impairment: a resting-state fMRI study. Front. Hum. Neurosci. 9:471. 10.3389/fnhum.2015.00471 26379534 PMC4550786

[B25] Chen K. Reiman E. M. Alexander G. E. Caselli R. J. Gerkin R. Bandy D.. (2007). Correlations between apolipoprotein E ε4 gene dose and whole brain atrophy rates. Am. J. Psychiatry 164, 916–921. 10.1176/ajp.2007.164.6.916 17541051

[B7] Chien D. T. Szardenings A. K. Bahri S. Walsh J. C. Mu F. Xia C.. (2014). Early clinical PET imaging results with the novel PHF-tau radioligand [F18]-T808. J. Alzheimers Dis. 38, 171–184. 10.3233/jad-130098 23948934

[B8] Cho H. Choi J. Y. Mi S. H. Lee J. H. Kim Y. J. Lee H. M.. (2016). Tau PET in Alzheimer disease and mild cognitive impairment. Neurology 87, 375–383. 10.1212/WNL.0000000000002892 27358341

[B9] Cope T. E. Rittman T. Borchert R. J. Jones P. S. Vatansever D. Allinson K.. (2018). Tau burden and the functional connectome in Alzheimer’s disease and progressive supranuclear palsy. Brain 141, 550–567. 10.1093/brain/awx347 29293892 PMC5837359

[B10] Danon L. Díaz-Guilera A. Duch J. Arenas A. (2005). Comparing community structure identification. J. Stat. Mech. Theory Exp. 2005:P09008 10.1088/1742-5468/2005/09/p09008

[B11] de Toledo-Morrell L. Goncharova I. Dickerson B. Wilson R. S. Bennett D. A. (2010). From healthy aging to early Alzheimer’s disease: *in vivo* detection of entorhinal cortex atrophy. Ann. N Y Acad. Sci. 911, 240–253. 10.1111/j.1749-6632.2000.tb06730.x 10911878

[B12] Duan H. Jiang J. Xu J. Zhou H. Huang Z. Yu Z.. (2017). Differences in Aβ brain networks in Alzheimer’s disease and healthy controls. Brain Res. 1655, 77–89. 10.1016/j.brainres.2016.11.019 27867033

[B13] Dyrba M. Mohammadi R. Grothe M. J. Kirste T. Teipel S. J. (2018). Assessing inter-modal and inter-regional dependencies in prodromal Alzheimer’s disease using multimodal MRI/PET and Gaussian graphical models. arXiv [Preprint arXiv:1804.00049].

[B16] Fennema-Notestine C. Panizzon M. S. Thompson W. R. Chen C. H. Eyler L. T. Fischl B.. (2011). Presence of ApoE ε4 allele associated with thinner frontal cortex in middle age. J. Alzheimers Dis. 26, 49–60. 10.3233/JAD-2011-0002 21971450 PMC3302177

[B17] Filippini N. Rao A. Wetten S. Gibson R. A. Borrie M. Guzman D.. (2009). Anatomically-distinct genetic associations of *APOE* ε4 allele load with regional cortical atrophy in Alzheimer’s disease. Neuroimage 44, 724–728. 10.1016/j.neuroimage.2008.10.003 19013250

[B24] Fortea J. Vilaplana E. Alcolea D. Carmona-Iragui M. Sánchez-Saudinos M. B. Sala I.. (2014). Cerebrospinal fluid β-amyloid and phospho-tau biomarker interactions affecting brain structure in preclinical Alzheimer disease. Ann. Neurol. 76, 223–230. 10.1002/ana.24186 24852682

[B18] Fox N. C. Warrington E. K. Freeborough P. A. Hartikainen P. Kennedy A. M. Stevens J. M.. (1996). Presymptomatic hippocampal atrophy in Alzheimer’s disease. A longitudinal MRI study. Brain 119, 2001–2007. 10.1093/brain/119.6.2001 9010004

[B19] Gabrieli J. D. E. Desmond J. E. Demb J. B. Wagner A. D. Stone M. V. Vaidya C. J. (1996). Functional magnetic resonance imaging of semantic memory processes in the frontal lobes. Psychol. Sci. 7, 278–283. 10.1111/j.1467-9280.1996.tb00374.x

[B20] Gispert J. D. Monté G. C. Falcon C. Tucholka A. Rojas S. Sánchez-Valle R.. (2016). CSF YKL-40 and pTau181 are related to different cerebral morphometric patterns in early AD. Neurobiol. Aging 38, 47–55. 10.1016/j.neurobiolaging.2015.10.022 26827642

[B21] Goryawala M. Duara R. Loewenstein D. A. Zhou Q. Barker W. Adjouadi M. (2015). Apolipoprotein-E4 (ApoE4) carriers show altered small-world properties in the default mode network of the brain. Biomed. Phys. Eng. Exp. 1:015001 10.1088/2057-1976/1/1/015001

[B22] Haense C. Buerger K. Kalbe E. Drzezga A. Teipel S. J. Markiewicz P.. (2008). CSF total and phosphorylated tau protein, regional glucose metabolism and dementia severity in Alzheimer’s disease. Eur. J. Neurol. 15, 1155–1162. 10.1111/j.1468-1331.2008.02274.x 18803648

[B23] Honea R. A. Vidoni E. Harsha A. Burns J. M. (2009). Impact of *APOE* on the healthy aging brain: a voxel-based MRI and DTI study. J. Alzheimers Dis. 18, 553–564. 10.3233/jad-2009-1163 19584447 PMC2892293

[B34] Lehmann M. Ghosh P. M. Madison C. Karydas A. Coppola G. O’Neil J. P.. (2014). Greater medial temporal hypometabolism and lower cortical amyloid burden in ApoE4-positive AD patients. J. Neurol. Neurosurg. Psychiatry 85, 266–273. 10.1136/jnnp-2013-305858 23965289 PMC3946299

[B27] Lehtovirta M. Laakso M. P. Soininen H. Helisalmi S. Mannermaa A. Helkala E. L.. (1995). Volumes of hippocampus, amygdala and frontal lobe in Alzheimer patients with different apolipoprotein E genotypes. Neuroscience 67, 65–72. 10.1016/0306-4522(95)00014-a 7477910

[B28] Lehtovirta M. Soininen H. Laakso M. Partanen K. Helisalmi S. Mannermaa A.. (1996). SPECT and MRI analysis in Alzheimer’s disease: relation to apolipoprotein E ε 4 allele. J. Neurol. Neurosurg. Psychiatry 60, 644–649. 10.1136/jnnp.60.6.644 8648331 PMC1073948

[B29] Leritz E. C. Shepel J. Williams V. J. Lipsitz L. A. McGlinchey R. E. Milberg W. P.. (2014). Associations between T1 white matter lesion volume and regional white matter microstructure in aging. Hum. Brain Mapp. 35, 1085–1100. 10.1002/hbm.22236 23362153 PMC4356252

[B30] Leube D. T. Weis S. Freymann K. Erb M. Jessen F. Heun R.. (2008). Neural correlates of verbal episodic memory in patients with MCI and Alzheimer’s disease—a VBM study. Int. J. Geriatr. Psychiatry 23, 1114–1118. 10.1002/gps.2036 18449954

[B31] Li W. Antuono P. G. Xie C. Chen G. Jones J. L. Ward B. D.. (2014). Aberrant functional connectivity in Papez circuit correlates with memory performance in cognitively intact middle-aged *APOE*4 carriers. Cortex 57, 167–176. 10.1016/j.cortex.2014.04.006 24905971 PMC4448757

[B32] Liu C. Gao J. Niu N. (2013). The dynamic changing of occipital dementia and posterior cortical atrophy. Alzheimers Dement. 9, P748–P749. 10.1016/j.jalz.2013.05.1513

[B33] Lo C. Y. Wang P. N. Chou K. H. Wang J. He Y. Lin C. P. (2010). Diffusion tensor tractography reveals abnormal topological organization in structural cortical networks in Alzheimer’s disease. J. Neurosci. 30, 16876–16885. 10.1523/JNEUROSCI.4136-10.2010 21159959 PMC6634928

[B35] Marquié M. Normandin M. D. Vanderburg C. R. Costantino I. M. Bien E. A. Rycyna L. G.. (2015). Validating novel tau positron emission tomography tracer [F-18]-AV-1451 (T807) on postmortem brain tissue. Ann. Neurol. 78, 787–800. 10.1002/ana.24517 26344059 PMC4900162

[B39] Mattsson N. Zetterberg H. Hansson O. Andreasen N. Parnetti L. Jonsson H.. (2009). CSF biomarkers and incipient Alzheimer disease in patients with mild cognitive impairment. JAMA 302, 385–393. 10.1001/jama.2009.1064 19622817

[B36] Newman M. E. (2006). Finding community structure in networks using the eigenvectors of matrices. Phys. Rev. E Stat. Nonlin. Soft Matter Phys. 74:036104. 10.1103/physreve.74.036104 17025705

[B38] Newman M. E. Girvan M. (2004). Finding and evaluating community structure in networks. Phys. Rev. E Stat. Nonlin. Soft Matter Phys. 69:026113. 10.1103/PhysRevE.69.026113 14995526

[B40] Pereira J. B. Mijalkov M. Kakaei E. Mecocci P. Vellas B. Tsolaki M.. (2016). Disrupted network topology in patients with stable and progressive mild cognitive impairment and Alzheimer’s disease. Cereb. Cortex 26, 3476–3493. 10.1093/cercor/bhw128 27178195 PMC4961019

[B41] Pereira J. B. van Westen D. Stomrud E. Strandberg T. O. Volpe G. Westman E.. (2018). Abnormal structural brain connectome in individuals with preclinical Alzheimer’s disease. Cereb. Cortex 28, 3638–3649. 10.1093/cercor/bhx236 29028937

[B42] Phelps E. A. (2006). Emotion and cognition: insights from studies of the human amygdala. Annu. Rev. Psychol. 57, 27–53. 10.1146/annurev.psych.56.091103.070234 16318588

[B43] Poulin S. P. Dautoff R. Morris J. C. Barrett L. F. Dickerson B. C. Alzheimer’s Disease Neuroimaging Initiative. (2011). Amygdala atrophy is prominent in early Alzheimer’s disease and relates to symptom severity. Psychiatry Res. 194, 7–13. 10.1016/j.pscychresns.2011.06.014 21920712 PMC3185127

[B44] Rubinov M. Sporns O. (2010). Complex network measures of brain connectivity: uses and interpretations. Neuroimage 52, 1059–1069. 10.1016/j.neuroimage.2009.10.003 19819337

[B45] Sala-Llonch R. Bartrés-Faz D. Junqué C. (2015). Reorganization of brain networks in aging: a review of functional connectivity studies. Front. Psychol. 6:663. 10.3389/fpsyg.2015.00663 26052298 PMC4439539

[B46] Salvatore C. Battista P. Castiglioni I. (2016). Frontiers for the early diagnosis of AD by means of MRI brain imaging and support vector machines. Curr. Alzheimer Res. 13, 509–533. 10.2174/1567205013666151116141705 26567735

[B47] Sanabria-Diaz G. Martínez-Montes E. Melie-Garcia L. Alzheimer’s Disease Neuroimaging Initiative. (2013). Glucose metabolism during resting state reveals abnormal brain networks organization in the Alzheimer’s disease and mild cognitive impairment. PLoS One 8:e68860. 10.1371/journal.pone.0068860 23894356 PMC3720883

[B14] Seo E. H. Lee D. Y. Lee J. M. Park J. S. Sohn B. K. Lee D. S.. (2013). Whole-brain functional networks in cognitively normal, mild cognitive impairment, and Alzheimer’s disease. PLoS One 8:e53922. 10.1371/journal.pone.0053922 23335980 PMC3545923

[B48] Schöll M. Ossenkoppele R. Strandberg O. Palmqvist S. The Swedish BioFINDER study Jögi J.. (2017). Distinct 18F-AV-1451 tau PET retention patterns in early-and late-onset Alzheimer’s disease. Brain 140, 2286–2294. 10.1093/brain/awx171 29050382

[B49] Schwarz A. J. Yu P. Miller B. B. Shcherbinin S. Dickson J. Navitsky M.. (2016). Regional profiles of the candidate tau PET ligand 18 F-AV-1451 recapitulate key features of Braak histopathological stages. Brain 139, 1539–1550. 10.1093/brain/aww023 26936940

[B50] Shaw L. M. Vanderstichele H. Knapik-Czajka M. Clark C. M. Aisen P. S. Petersen R. C.. (2009). Cerebrospinal fluid biomarker signature in Alzheimer’s disease neuroimaging initiative subjects. Ann. Neurol. 65, 403–413. 10.1002/ana.21610 19296504 PMC2696350

[B52] Sporns O. Chialvo D. R. Kaiser M. Hilgetag C. C. (2004). Organization, development and function of complex brain networks. Trends Cogn. Sci. 8, 418–425. 10.1016/j.tics.2004.07.008 15350243

[B51] Sporns O. Zwi J. D. (2004). The small world of the cerebral cortex. Neuroinformatics 2, 145–162. 10.1385/NI:2:2:145 15319512

[B54] Stam C. J. Jones B. F. Nolte G. Breakspear M. Scheltens P. (2007). Small-world networks and functional connectivity in Alzheimer’s disease. Cereb. Cortex 17, 92–99. 10.1093/cercor/bhj127 16452642

[B55] Teipel S. J. (2010). DTI and resting state fMRI as biomarker of Alzheimer’s disease: present state and perspectives. Alzheimers Dement. 6:S169 10.1016/j.jalz.2010.05.527

[B26] Tsapkini K. Frangakis C. E. Hillis A. E. (2011). The function of the left anterior temporal pole: evidence from acute stroke and infarct volume. Brain 134, 3094–3105. 10.1093/brain/awr050 21685458 PMC3187536

[B56] Unterrainer J. M. Owen A. M. (2006). Planning and problem solving: from neuropsychology to functional neuroimaging. J. Physiol. Paris 99, 308–317. 10.1016/j.jphysparis.2006.03.014 16750617

[B57] van der Berg W. Zweekhorst S. Voorn P. Groenewegen H. Hoogland P. Rozemuller A. M. (2007). Pattern of α-synuclein and phosphorylated tau pathology in the olfactory bulb, brainstem and limbic regions in aged individuals. Parkinsonism Relat. Disord. 13:S122 10.1016/s1353-8020(08)70701-6

[B15] Vecchio F. Miraglia F. Curcio G. Altavilla R. Scrascia F. Giambattistelli F.. (2015). Cortical brain connectivity evaluated by graph theory in dementia: a correlation study between functional and structural data. J. Alzheimers Dis. 45, 745–756. 10.3233/JAD-142484 25613102

[B58] Villain N. Fouquet M. Baron J.-C. Mézenge F. Landeau B. de La Sayette V.. (2010). Sequential relationships between grey matter and white matter atrophy and brain metabolic abnormalities in early Alzheimer’s disease. Brain 133, 3301–3314. 10.1093/brain/awq203 20688814 PMC3291528

[B59] Waldemar G. Dubois B. Emre M. Georges J. Mckeith I. G. Rossor M.. (2007). Recommendations for the diagnosis and management of Alzheimer’s disease and other disorders associated with dementia: EFNS guideline. Eur. J. Neurol. 14, e1–e26. 10.1111/j.1468-1331.2006.01605.x 17222085

[B60] Weaver C. L. Espinoza M. Kress Y. Davies P. (2000). Conformational change as one of the earliest alterations of tau in Alzheimer’s disease. Neurobiol. Aging 21, 719–727. 10.1016/s0197-4580(00)00157-3 11016541

[B61] Yao Z. Hu B. Chen X. Xie Y. Gutknecht J. Majoe D. (2018). Learning metabolic brain networks in MCI and AD by robustness and leave-one-out analysis: an FDG-PET study. Am. J. Alzheimers Dis. Other Demen. 33, 42–54. 10.1177/1533317517731535 28931302 PMC10852436

[B62] Yao Z. Hu B. Zheng J. Zheng W. Chen X. Gao X.. (2015). A FDG-PET study of metabolic networks in apolipoprotein E ε4 allele carriers. PLoS One 10:e0132300. 10.1371/journal.pone.0132300 26161964 PMC4498596

[B63] Yao Z. Zhang Y. Lin L. Zhou Y. Xu C. Jiang T.. (2010). Abnormal cortical networks in mild cognitive impairment and Alzheimer’s disease. PLoS Comput. Biol. 6:e1001006. 10.1371/journal.pcbi.1001006 21124954 PMC2987916

[B64] Zhang H. Ng K. P. Therriault J. Kang M. S. Pascoal T. A. Rosa-Neto P.. (2018). Cerebrospinal fluid phosphorylated tau, visinin-like protein-1 and chitinase-3-like protein 1 in mild cognitive impairment and Alzheimer’s disease. Transl. Neurodegener. 7:23. 10.1186/s40035-018-0127-7 30311914 PMC6161434

[B66] Zhao X. Liu Y. Wang X. Liu B. Xi Q. Guo Q.. (2012). Disrupted small-world brain networks in moderate Alzheimer’s disease: a resting-state FMRI study. PLoS One 7:e33540. 10.1371/journal.pone.0033540 22457774 PMC3311642

[B65] Zhao F. Zhang H. Rekik I. An Z. Shen D. (2018). Diagnosis of autism spectrum disorders using multi-level high-order functional networks derived from resting-state functional MRI. Front. Hum. Neurosci. 12:184. 10.3389/fnhum.2018.00184 29867410 PMC5960713

[B67] Zheng W. Eilamstock T. Wu T. Spagna A. Fan J. Chen C. Hu B. (2019). Multi-feature based network revealing the structural abnormalities in autism spectrum disorder. IEEE Transactions on Affective Computing, 1–1. 10.1109/TAFFC.2018.2890597

