# Supplementary material for: The Influence of Cerebrospinal Fluid Abnormalities and APOE 4 on PHF-Tau Protein: Evidence From Voxel Analysis and Graph Theory
Source: Front Aging Neurosci. 2019 Aug 8;11:208. doi: 10.3389/fnagi.2019.00208 (PMC6694441; doi:10.3389/fnagi.2019.00208)
Supplement: Supplementary file 1 [file Data_Sheet_1.docx]

**Supplementary information**

The results of regional and global correlations between CSF Tau and PET.

Table S1: Regional correlations between CSF T-Tau and Tau-PET.

| **Regions** |  | P value |  |
| --- | --- | --- | --- |
|  | APOE 4+T+ | APOE 4+T- | APOE 4-T+ |
| PreCG.L | 0.338 | 0.336 | 0.997 |
| PreCG.R | 0.221 | 0.337 | 0.947 |
| SFGdor.L | 0.225 | 0.333 | 0.256 |
| SFGdor.R | 0.151 | 0.282 | 0.256 |
| ORBsup.L | 0.252 | 0.324 | 0.199 |
| ORBsup.R | 0.096 | 0.246 | 0.163 |
| MFG.L | 0.456 | 0.348 | 0.193 |
| MFG.R | 0.263 | 0.373 | 0.347 |
| ORBmid.L | 0.518 | 0.338 | 0.202 |
| ORBmid.R | 0.232 | 0.284 | 0.178 |
| IFGoperc.L | 0.302 | 0.28 | 0.466 |
| IFGoperc.R | 0.124 | 0.445 | 0.566 |
| IFtriang.L | 0.489 | 0.37 | 0.304 |
| IFtriang.R | 0.340 | 0.599 | 0.419 |
| ORBinf.L | 0.351 | 0.264 | 0.325 |
| ORBinf.R | 0.274 | 0.395 | 0.31 |
| ROL.L | 0.133 | 0.328 | 0.309 |
| ROL.R | 0.074 | 0.363 | 0.363 |
| SMA.L | 0.077 | 0.308 | 0.467 |
| SMA.R | 0.069 | 0.303 | 0.486 |
| OLF.L | 0.089 | 0.278 | 0.351 |
| OLF.R | **0.042** | 0.284 | 0.431 |
| SFGmed.L | 0.156 | 0.253 | 0.32 |
| SFGmed.R | 0.130 | 0.277 | 0.218 |
| ORBsupmed.L | 0.206 | 0.264 | 0.197 |
| ORBsupmed.R | 0.145 | 0.244 | 0.175 |
| REC.L | 0.165 | 0.32 | 0.176 |
| REC.R | 0.092 | 0.248 | 0.161 |
| INS.L | 0.252 | 0.419 | 0.281 |
| INS.R | 0.075 | 0.365 | 0.245 |
| ACG.L | 0.175 | 0.233 | 0.497 |
| ACG.R | 0.120 | 0.245 | 0.541 |
| DCG.L | 0.263 | 0.298 | 0.693 |
| DCG.R | 0.241 | 0.309 | 0.834 |
| PCG.L | 0.326 | 0.248 | 0.624 |
| PCG.R | 0.301 | 0.213 | 0.921 |
| HIP.L | 0.124 | 0.495 | 0.829 |
| HIP.R | 0.184 | 0.413 | 0.999 |
| PHG.L | 0.12 | 0.657 | 0.162 |
| PHG.R | **0.045** | 0.609 | 0.251 |
| AMYG.L | 0.162 | 0.782 | 0.258 |
| AMYG.R | 0.165 | 0.565 | 0.464 |
| CAL.L | 0.458 | 0.412 | 0.517 |
| CAL.R | 0.221 | 0.452 | 0.358 |
| CUN.L | 0.473 | 0.303 | 0.679 |
| CUN.R | 0.336 | 0.428 | 0.520 |
| LING.L | 0.426 | 0.431 | 0.19 |
| LING.R | 0.185 | 0.503 | 0.254 |
| SOG.L | 0.706 | 0.300 | 0.658 |
| SOG.R | 0.340 | 0.459 | 0.524 |
| MOG.L | 0.678 | 0.379 | 0.318 |
| MOG.R | 0.276 | 0.477 | 0.329 |
| IOG.L | 0.772 | 0.439 | 0.072 |
| IOG.R | 0.322 | 0.603 | 0.181 |
| FFG.L | 0.237 | 0.600 | 0.068 |
| FFG.R | 0.147 | 0.579 | 0.110 |
| PoCG.L | 0.307 | 0.284 | 0.818 |
| PoCG.R | 0.205 | 0.322 | 0.909 |
| SPG.L | 0.465 | 0.329 | 0.669 |
| SPG.R | 0.554 | 0.301 | 0.994 |
| IPL.L | 0.346 | 0.301 | 0.374 |
| IPL.R | 0.134 | 0.29 | 0.616 |
| SMG.L | 0.421 | 0.336 | 0.463 |
| SMG.R | 0.175 | 0.354 | 0.751 |
| ANG.L | 0.771 | 0.303 | 0.477 |
| ANG.R | 0.391 | 0.268 | 0.744 |
| PCUN.L | 0.276 | 0.344 | 0.429 |
| PCUN.R | 0.262 | 0.338 | 0.594 |
| PCL.L | 0.133 | 0.486 | 0.880 |
| PCL.R | 0.186 | 0.481 | 0.773 |
| CAU.L | 0.168 | 0.197 | 0.621 |
| CAU.R | 0.20 | 0.187 | 0.559 |
| PUT.L | 0.541 | 0.521 | 0.554 |
| PUT.R | 0.219 | 0.504 | 0.402 |
| PAL.L | 0.747 | 0.520 | 0.906 |
| PAL.R | 0.667 | 0.517 | 0.958 |
| THA.L | 0.376 | 0.403 | 0.937 |
| THA.R | 0.477 | 0.372 | 0.978 |
| HES.L | 0.244 | 0.422 | 0.161 |
| HES.R | 0.118 | 0.323 | 0.127 |
| STG.L | 0.261 | 0.447 | 0.098 |
| STG.R | 0.157 | 0.402 | 0.151 |
| TPOsup.L | 0.332 | 0.374 | 0.353 |
| TPOsup.R | 0.274 | 0.43 | 0.505 |
| MTG.L | 0.465 | 0.435 | 0.115 |
| MTG.R | 0.185 | 0.417 | 0.17 |
| TPOmid.L | 0.215 | 0.838 | 0.292 |
| TPOmid.R | 0.153 | 0.942 | 0.394 |
| ITG.L | 0.317 | 0.544 | 0.106 |
| ITG.R | 0.169 | 0.567 | 0.116 |

Table S2: Global correlations between CSF T-Tau and Tau-PET

|  | P value |  |
| --- | --- | --- |
| APOE 4+T+ | APOE 4+T- | APOE 4-T+ |
| 0.205 | 0.364 | 0.222 |

Table S3: Regional correlations between CSF P-Tau and Tau-PET.

| **Regions** |  | P value |  |
| --- | --- | --- | --- |
|  | APOE 4+T+ | APOE 4+T- | APOE 4-T+ |
| PreCG.L | 0.282 | 0.134 | 0.965 |
| PreCG.R | 0.192 | 0.163 | 0.889 |
| SFGdor.L | 0.182 | 0.161 | 0.304 |
| SFGdor.R | 0.13 | 0.15 | 0.303 |
| ORBsup.L | 0.184 | 0.172 | 0.188 |
| ORBsup.R | 0.0591 | 0.126 | 0.145 |
| MFG.L | 0.406 | 0.162 | 0.234 |
| MFG.R | 0.246 | 0.203 | 0.42 |
| ORBmid.L | 0.423 | 0.169 | 0.204 |
| ORBmid.R | 0.189 | 0.129 | 0.174 |
| IFGoperc.L | 0.262 | 0.117 | 0.539 |
| IFGoperc.R | 0.118 | 0.224 | 0.689 |
| IFtriang.L | 0.461 | 0.166 | 0.39 |
| IFtriang.R | 0.346 | 0.32 | 0.544 |
| ORBinf.L | 0.297 | 0.127 | 0.37 |
| ORBinf.R | 0.239 | 0.182 | 0.369 |
| ROL.L | 0.112 | 0.148 | 0.415 |
| ROL.R | 0.0668 | 0.183 | 0.483 |
| SMA.L | 0.0655 | 0.148 | 0.502 |
| SMA.R | 0.0769 | 0.153 | 0.492 |
| OLF.L | 0.06 | 0.168 | 0.387 |
| OLF.R | 0.0242 | 0.159 | 0.478 |
| SFGmed.L | 0.137 | 0.0995 | 0.438 |
| SFGmed.R | 0.127 | 0.118 | 0.305 |
| ORBsupmed.L | 0.147 | 0.132 | 0.23 |
| ORBsupmed.R | 0.0966 | 0.122 | 0.21 |
| REC.L | 0.114 | 0.187 | 0.172 |
| REC.R | 0.053 | 0.143 | 0.152 |
| INS.L | 0.206 | 0.216 | 0.353 |
| INS.R | 0.059 | 0.207 | 0.328 |
| ACG.L | 0.137 | 0.091 | 0.631 |
| ACG.R | 0.0955 | 0.095 | 0.658 |
| DCG.L | 0.204 | 0.133 | 0.703 |
| DCG.R | 0.19 | 0.131 | 0.838 |
| PCG.L | 0.275 | 0.12 | 0.576 |
| PCG.R | 0.27 | 0.0973 | 0.919 |
| HIP.L | 0.106 | 0.253 | 0.707 |
| HIP.R | 0.162 | 0.215 | 0.831 |
| PHG.L | 0.0725 | 0.425 | 0.121 |
| PHG.R | 0..82 | 0.405 | 0.19 |
| AMYG.L | 0.12 | 0.537 | 0.201 |
| AMYG.R | 0.115 | 0.372 | 0.403 |
| CAL.L | 0.478 | 0.304 | 0.605 |
| CAL.R | 0.227 | 0.298 | 0.423 |
| CUN.L | 0.457 | 0.171 | 0.75 |
| CUN.R | 0.303 | 0.224 | 0.574 |
| LING.L | 0.369 | 0.294 | 0.191 |
| LING.R | 0.14 | 0.37 | 0.27 |
| SOG.L | 0.692 | 0.172 | 0.715 |
| SOG.R | 0.294 | 0.258 | 0.565 |
| MOG.L | 0.601 | 0.236 | 0.325 |
| MOG.R | 0.226 | 0.312 | 0.372 |
| IOG.L | 0.656 | 0.327 | 0.0651 |
| IOG.R | 0.227 | 0.529 | 0.194 |
| FFG.L | 0.148 | 0.405 | 0.0444 |
| FFG.R | 0.0786 | 0.403 | 0.085 |
| PoCG.L | 0.249 | 0.106 | 0.88 |
| PoCG.R | 0.169 | 0.156 | 0.987 |
| SPG.L | 0.377 | 0.172 | 0.679 |
| SPG.R | 0.488 | 0.181 | 0.962 |
| IPL.L | 0.257 | 0.133 | 0.35 |
| IPL.R | 0.094 | 0.176 | 0.649 |
| SMG.L | 0.324 | 0.116 | 0.474 |
| SMG.R | 0.13 | 0.158 | 0.811 |
| ANG.L | 0.657 | 0.13 | 0.455 |
| ANG.R | 0.32 | 0.139 | 0.786 |
| PCUN.L | 0.221 | 0.18 | 0.403 |
| PCUN.R | 0.214 | 0.176 | 0.57 |
| PCL.L | 0.0992 | 0.222 | 0.902 |
| PCL.R | 0.134 | 0.22 | 0.728 |
| CAU.L | 0.208 | 0.07 | 0.739 |
| CAU.R | 0.242 | 0.0757 | 0.678 |
| PUT.L | 0.552 | 0.302 | 0.597 |
| PUT.R | 0.207 | 0.305 | 0.442 |
| PAL.L | 0.774 | 0.262 | 0.871 |
| PAL.R | 0.691 | 0.279 | 0.946 |
| THA.L | 0.467 | 0.187 | 0.993 |
| THA.R | 0.578 | 0.18 | 0.947 |
| HES.L | 0.233 | 0.238 | 0.236 |
| HES.R | 0.125 | 0.203 | 0.215 |
| STG.L | 0.191 | 0.24 | 0.131 |
| STG.R | 0.127 | 0.237 | 0.221 |
| TPOsup.L | 0.23 | 0.214 | 0.384 |
| TPOsup.R | 0.215 | 0.234 | 0.575 |
| MTG.L | 0.348 | 0.268 | 0.107 |
| MTG.R | 0.138 | 0.290 | 0.187 |
| TPOmid.L | 0.117 | 0.544 | 0.286 |
| TPOmid.R | 0.093 | 0.690 | 0.371 |
| ITG.L | 0.202 | 0.376 | 0.067 |
| ITG.R | 0.099 | 0.417 | 0.097 |

Table S4: Global correlations between CSF P-Tau and Tau-PET.

|  | P value |  |
| --- | --- | --- |
| APOE 4+T+ | APOE 4+T- | APOE 4-T+ |
| 0.162 | 0.418 | 0.188 |
